# Critical care outcomes, for the first 200 patients with confirmed COVID-19, in England, Wales and Northern Ireland: A report from the ICNARC Case Mix Programme

**DOI:** 10.1177/1751143720961672

**Published:** 2021-11

**Authors:** Alvin Richards-Belle, Izabella Orzechowska, James Doidge, Karen Thomas, David A Harrison, Abby Koelewyn, Michael D Christian, Manu Shankar-Hari, Kathryn M Rowan, Doug W Gould

**Affiliations:** 1 Intensive Care National Audit and Research Centre (ICNARC), London, UK; 2 London’s Air Ambulance, Barts Health NHS Trust, The Royal London Hospital, London, UK; 3 Intensive Care Unit, St Thomas’ Hospital, Guy’s and St Thomas’ NHS Foundation Trust, London, UK

**Keywords:** COVID-19, coronavirus, intensive care, outcomes

## Abstract

**Background:**

Early in a pandemic, outcomes are biased towards patients with shorter durations of critical illness. We describe 60-day outcomes for patients critically ill with confirmed COVID-19 and explore the potential bias in the weekly reported data by ICNARC.

**Methods:**

First 200 consecutive patients with confirmed COVID-19, admitted for critical care in England, Wales and Northern Ireland, followed-up for a minimum of 60 days from admission. Outcomes included survival and duration of critical care, receipt/duration of organ support in critical care and hospital survival*.*

**Results:**

Mean age was 62.6 years, 70.5% were male, 52.0% were white, 39.2% obese and 9.0% had serious comorbidities. Median APACHE II score was 16 (IQR 12, 19). After 60 days, 83 (41.5%) patients had been discharged from hospital, 15 (7.5%) had been discharged from critical care but remained in hospital, 1 (0.5%) was still receiving critical care, 90 (45.0%) had died while receiving critical care and 11 (5.5%) had died in hospital after discharge from critical care. Median duration of critical care was 14.0 days (IQR 6.1, 23.0) for survivors and 10.0 days (IQR 5.0, 16.0) for non-survivors of critical care. Overall, 158 (79.0%) patients received advanced respiratory support for a median of 13 (IQR 8, 20) calendar days. Compared with weekly reports during the pandemic, critical care mortality started higher than but then decreased below that of the first 200 consecutive patients. Duration of critical care, for both survivors and non-survivors increased over time; however, both were still lower than those for the first 200 consecutive patients. Receipt and duration of organ support increased to values similar to those for the first 200 consecutive patients.

**Conclusion:**

COVID-19 in critical care has high mortality and places a large burden on resources. Analysis of preliminary data with limited follow-up should be interpreted with caution, particularly for future planning in a pandemic.

## Introduction

In late 2019, an outbreak of a novel zoonotic coronavirus infection (severe acute respiratory syndrome coronavirus 2) began to emerge in humans with its epicentre in Wuhan, China.^1,2^ On 11 February 2020, the WHO announced “COVID-19” as the name for this new disease^3^ and, on 11 March 2020, the WHO declared a COVID-19 pandemic.^4^ The first cases of COVID-19 were reported in the United Kingdom (UK) in late January 2020 and, as of 22 May 2020, the number of tested positive cases was 254,195 associated with 36,393 deaths.^5^

To help inform planning of critical care services, both centrally and locally, the Intensive Care National Audit & Research Centre (ICNARC), was well placed to rapidly collate, analyse and report data, weekly, on patients critically ill with confirmed COVID-19 by virtue of its co-ordination of the Case Mix Programme (CMP), the national clinical audit for adult critical care covering England, Wales and Northern Ireland. Commencing Friday 20 March, ICNARC circulated, and posted on its website, weekly analyses of data on patients critically ill with confirmed COVID-19.

Due to the gradual escalation of the UK epidemic and anecdotal evidence of long critical care stays for some patients with COVID-19, it was anticipated that the weekly analysis of patient outcomes reported by ICNARC might be biased towards those with shorter lengths of stay. This paper presents a new analysis of 60-day outcomes for the first 200 consecutive patients critically ill with confirmed COVID-19 in England, Wales and Northern Ireland and explores the potential bias in the ICNARC weekly reports.

## Methods

### Design

A prospective cohort of patients, critically ill with confirmed COVID-19, admitted to critical care units participating in the CMP.

### Sites and patients

The first 200 consecutive patients identified from their first admission with confirmed COVID-19 (confirmed either at or after the start of critical care), to one of 285 NHS adult critical care units in England, Wales and Northern Ireland (100% coverage) routinely submitting data to the CMP. Confirmed COVID-19 was defined as either a positive test (according to local hospital practice) or a clinical diagnosis of COVID-19 in the context of a negative test where the treating clinical team were convinced that the test was a false negative and the patient was treated as a COVID-19 patient.

### Data

As the UK epidemic emerged, relevant staff at CMP units were requested to notify ICNARC of any admission critically ill with confirmed COVID-19 and to submit data characterising the admission at the end of the first 24 h in the unit. At discharge from the unit, data summarising type and duration of organ system support and outcome from critical care were also provided.

Age, sex and ethnicity, the latter using NHS ethnic category codes, were recorded. Body mass index (BMI) was calculated from actual measurements of height and weight (or estimated measurements, where actual not available). Data were recorded for prior duration of stay (in hospital) and source of admission to the critical care unit. With respect to medical history, data collection covered: receipt (within 24 h prior to critical care admission) and location (community/in-hospital) of cardiopulmonary resuscitation (CPR); prior dependency based on levels of assistance with daily activities (e.g. daily activities include bathing, dressing, going to the toilet, moving in/out of bed/chair, continence and eating); and serious comorbidities. During the first 24 h in the critical care unit, lowest and highest values for physiological parameters, required for determination and calculation of acute illness severity, were also recorded.

Serious comorbidities, evident in the six months prior to admission, were defined as: cardiovascular – symptoms of fatigue, claudication, dyspnoea or angina at rest; respiratory – shortness of breath with light activity or home ventilation; renal – receipt of renal replacement therapy for end-stage renal disease; liver – biopsy-proven cirrhosis, portal hypertension or hepatic encephalopathy; metastatic disease – distant metastases; haematological malignancy – acute or chronic leukaemia, multiple myeloma or lymphoma; and immunocompromise – receipt of chemotherapy, radiotherapy or high-dose steroid (daily) treatment, HIV/AIDS or a congenital immune deficiency.

Patients were followed up until death or discharge from hospital or, if still in hospital, for a minimum of 60 days from date of admission to critical care. Dates and times of critical care admission and discharge, including any readmissions to critical care during the same hospital stay, were collected to calculate total duration of stay in critical care. Calendar days (00:00 to 23:59) of organ support (respiratory, cardiovascular, renal, neurological) in critical care, defined by the NHS Critical Care Minimum Data Set (CCMDS),^6^ were also collected.

All data were collected prospectively, and abstracted retrospectively, according to precise rules and definitions,^7^ as for the Case Mix Programme, under Section 251 of the NHS Act 2006 (approval number PIAG 2–10(f)/2005).

### Data management and statistical analysis

Age was derived from dates of birth and admission to critical care. Recorded ethnicity sub-codes were collapsed into five categories as: white (white-British, white-Irish, white-any other); Asian (Asian or Asian British-Indian, Asian or Asian British-Pakistani, Asian or Asian British-Bangladeshi, Asian or Asian British-any other); black (black or black British-Caribbean, black or black British-African, black or black British-any other); mixed/other (mixed-white and black Caribbean, mixed-white and black African, mixed-white and Asian, mixed-any other, other ethnic group-Chinese, and any other ethnic group); and not stated. BMI was calculated as weight (kilograms) divided by height (metres squared) and categorised into standard NHS BMI categories.

Prior hospital stay was calculated from dates of admission to acute hospital and to critical care. Source of admission to critical care was categorised as: emergency department; ward; other hospital location; or not in hospital. Prior dependency was considered in three categories: independent (those receiving no assistance with daily activities); some dependency (those receiving minor or major assistance with daily activities); and dependent (those receiving total assistance with daily activities).

Receipt of mechanical ventilation during the first 24 h was inferred from the recording of a ventilated respiratory rate. The PaO_2_/FiO_2_ ratio (P/F ratio), derived from the arterial blood gas with the lowest PaO_2_ during the first 24 h, was categorised to reflect mild, moderate and severe acute respiratory distress syndrome (ARDS): >200 mmHg (>26.7 kPa), >100 and ≤200 mmHg (>13.3 and ≤26.7 kPa) and ≤100 mmHg (≤13.3 kPa).^8^

The two acute severity scores, the ICNARC physiology score^9^ (0 to 100) and the Acute Physiology and Chronic Health Evaluation (APACHE) II^10^ acute physiology score (0 to 60), are based on weighting any deviation from the normal range for 12 physiological parameters during the first 24 h in the critical care unit. Both physiology scores weight temperature, heart rate, respiratory rate, arterial pH, serum sodium, serum creatinine, white blood cell count and Glasgow Coma Score. Additionally, the ICNARC physiology score weights systolic blood pressure, P/F ratio, serum urea and urine output, while the APACHE II acute physiology score weights mean arterial pressure, A-aDO2 (if FiO2 ≥0.5) or PaO2 (if FiO2 <0.5), serum potassium and haematocrit (estimated from haemoglobin). The APACHE II Score (0 to 71) adds additional weights for age and for serious comorbidities to the APACHE II acute physiology score.^8^

Subsequent admissions to critical care for COVID-19, for the same patient, were linked using NHS number, including both direct critical care transfers and readmissions to critical care within the same hospital stay. Patient characteristics presented derive from the first critical care admission. Total duration of stay in critical care was calculated from the dates and times of admission to and discharge from critical care, excluding any period in the hospital stay outside critical care.

Descriptive statistics were used to summarise data; results are reported as means with standard deviations (SD), medians with interquartile ranges (IQRs) or counts and percentages, as appropriate. Survival was analysed using a Kaplan–Meier curve with patients discharged alive from hospital treated as surviving until the end of the follow-up period.

Data were analysed as soon as all patients had completed follow-up. All analyses were conducted using Stata/SE version 14.2 (StataCorp LP).

### Patient and public involvement

No patients were involved in this descriptive analysis of the emerging epidemic.

## Results

### Sites and patients

**Figure 1.**
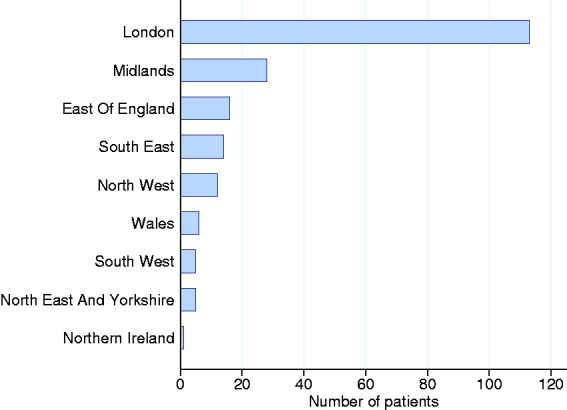
Number of patients by geographical region.

The first 200 consecutive patients, critically ill with confirmed COVID-19, were admitted to 96 of 285 participating critical care units between 20 February and 15 March 2020. Of these, 193 were treated in critical care units in England (113 in London), with six patients treated in Wales and one in Northern Ireland. The geographical spread of patient admissions is shown in Figure 1.

### Patient characteristics

Mean age was 62.6 (SD 13.4) years; with 7.0% aged under 40 and 8.0% aged over 80 years. Over two thirds of patients were male and 52.0% were white, 16.0% black and 18.0% Asian (7.0% mixed/other). Almost 40% were categorised as obese (BMI >30) (Table 1).

**Table 1.** Characteristics of patients critically ill with confirmed COVID-19.

Characteristic	*N*	Result
Demographics		
Age (years), mean (SD)	200	62.6 (13.4)
Age categories (years), *n* (%)	200	
16–29		4 (2.0%)
30–39		10 (5.0%)
40–49		18 (9.0%)
50–59		44 (22.0%)
60–69		61 (30.5%)
70–79		47 (23.5%)
80+		16 (8.0%)
Sex – Male, *n* (%)	200	141 (70.5%)
Ethnicity, *n* (%)	200	
White		104 (52.0%)
Asian		36 (18.0%)
Black		32 (16.0%)
Mixed/Other		14 (7.0%)
Not stated		14 (7.0%)
BMI (kg/m^2^), median (IQR)	196	28.6 (25.6,33.4)
BMI categories (kg/m^2^), *n* (%)	196	
<25		43 (21.9%)
25 to <30		76 (38.8%)
30 to <40		63 (32.1%)
40+		14 (7.1%)
Medical history		
Prior hospital stay (days), median (IQR)	200	1 (0,3)
Source of admission to critical care, *n* (%)	200	
Not in hospital		1 (0.5%)
Emergency department		66 (33.0%)
Ward		121 (60.5%)
Other hospital location^a^		12 (6.0%)
CPR within 24 h prior to admission to critical care, *n* (%)	200	
Community CPR		4 (2.0%)
In-hospital CPR		4 (2.0%)
None		192 (96.0%)
Prior dependency, *n* (%)	198	
Able to live without assistance in daily activities		162 (81.8%)
Some assistance with daily activities		35 (17.7%)
Total assistance with all daily activities		1 (0.5%)
Any serious comorbidities, n (%)^b^	200	18 (9.0%)
**Indicator of acute severity during first 24 h in critical care**		
Mechanical ventilation, *n* (%)	196	136 (69.4%)
Highest temperature (℃), mean (SD)	193	38.4 (1.1)
P/F ratio (kPa), median (IQR)^c^	186	15.1 (10.7, 21.9)
P/F ratio categories, *n* (%)	186	
≤13.3 kPa (≤100 mmHg)		72 (38.7%)
>13.3 and ≤26.7 kPa (>100 and ≤200 mmHg)		92 (49.5%)
>26.7 kPa (>200 mmHg)		22 (11.8%)
ICNARC physiology score,^d^ median (IQR)	200	18.5 (13, 23)
APACHE II acute physiology score,^e^ median (IQR)	198	12 (9, 15)
APACHE II score,^f^ median (IQR)	198	16 (12, 19)

Percentages may not total 100% owing to rounding.

BMI, body mass index; CPR, cardiopulmonary resuscitation; P/F ratio: PaO_2_/FiO_2_ ratio; ICNARC: Intensive Care National Audit & Research Centre; APACHE II: acute physiology and chronic health evaluation, second version.

a Other hospital location includes obstetrics areas, intermediate care areas, theatres, recovery, imaging departments, specialist treatment areas and clinics.

b Serious comorbidities are defined as: Cardiovascular: symptoms of fatigue, claudication, dyspnoea or angina at rest; Respiratory: shortness of breath with light activity or home ventilation; Renal: receipt of renal replacement therapy for end-stage renal disease; Liver: biopsy-proven cirrhosis, portal hypertension or hepatic encephalopathy; Metastatic disease: distant metastases; Haematological malignancy: acute or chronic leukaemia, multiple myeloma or lymphoma; and Immunocompromise: receipt of chemotherapy, radiotherapy or daily high-dose steroid treatment in previous 6 months, HIV/AIDS or a congenital immune deficiency.

c P/F ratio derived from the arterial blood gas with the lowest PaO_2_ during the first 24 h.

d ICNARC physiology score (range, 0–100; higher scores indicate greater severity) was calculated using physiological parameters recorded during the first 24 h in the critical care unit.

e APACHE II acute physiology score (range 0–60) was calculated using physiological parameters recorded during the first 24 h in the critical care unit.

f APACHE II score (range, 0–71; higher scores indicate greater severity) was calculated using the APACHE II acute physiology score plus weightings for age and serious comorbidities.

Prior hospital stay was short (median 1 day) and the majority of patients were admitted from either the ward (60.5%) or emergency department (33.0%). Very few (4.0%) received CPR within 24 h prior to admission for critical care. Most patients were reported as being previously independent; only 18.2% were reported as receiving at least some assistance with daily activities and only a small proportion (9.0%) had evidence of at least one, or more, of the serious comorbidities in the prior six months.

Almost 70% of patients were ventilated during the first 24 h, 60.1% experienced fever (defined as any temperature over 38℃) and almost 40% had P/F ratios equating to severe ARDS (Table 1). The median (IQR) APACHE II acute physiology score was 12 (9, 15), with a total APACHE II score of 16 (12, 19).

### Outcome, total duration of critical care and receipt and duration of organ support in critical care

**Figure 2.**
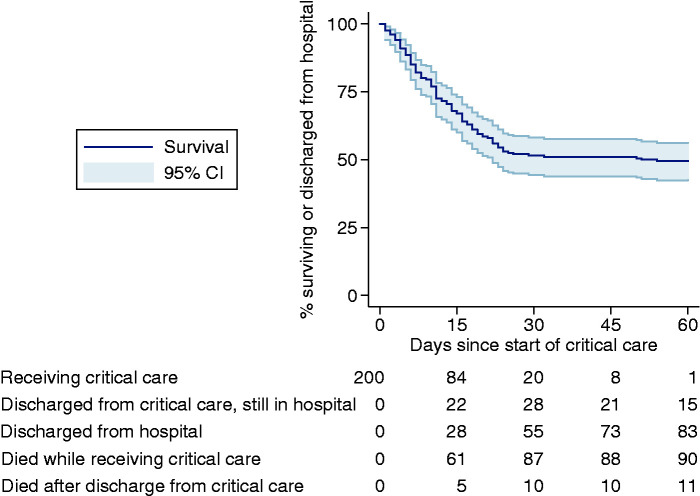
Kaplan–Meier analysis of survival to hospital discharge.

After 60 days, 83 (41.5%) patients had been discharged from hospital, 15 (7.5%) had been discharged from critical care but remained in hospital, 1 (0.5%) was still receiving critical care, 90 (45.0%) had died while receiving critical care and 11 (5.5%) had died in hospital after discharge from critical care. When data were extracted for analysis, a further three of the 16 patients still in hospital at 60 days had been discharged from hospital, one remained in critical care and none had died. The Kaplan-Meier survival curve is presented in Figure 2 and 60-day mortality was estimated to be 50.5% (CI 43.8, 57.6).

**Figure 3.**
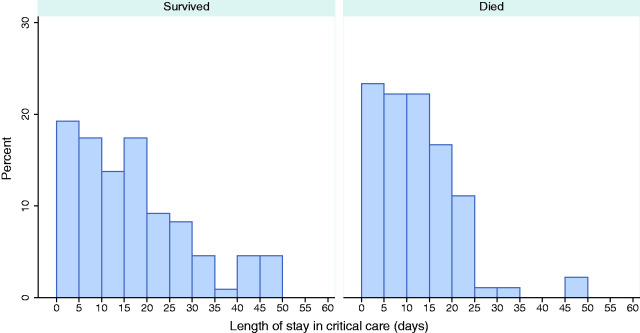
Total duration of critical care for critical care survivors and non-survivors. Distribution of time spent receiving critical care, combining transfers and readmissions (excluding any intervening periods) excluding one patient still receiving critical care. Denominators are the number of critical care survivors and non-survivors, respectively.

The median (IQR) total duration of critical care was 12 (5.5–19.9) days. Eight patients were readmitted to critical care including one patient who was readmitted twice. Three readmissions occurred within 48 h and six occurred between 48 h and five days after initial discharge from critical care. The median (IQR) total duration of critical care was 14 (IQR 6.1, 23) days for survivors and 10 (IQR 5, 16) days for non-survivors from critical care (Table 2). Distributions of total duration of critical care for survivors and non-survivors are presented in Figure 3. Advanced respiratory support was received by the majority (79.0%) of patients for a median of 13 (8, 20) calendar days. Fewer patients received advanced cardiovascular (40.5%), renal (31.0%) or neurological (10.6%) support and this support was given for shorter durations (Table 2). Almost all (95.2%) of those receiving renal support also received advanced respiratory support.

**Table 2.** Total duration of critical care and receipt and duration of organ support in critical care for patients critically ill with confirmed COVID-19.

	*N*	Result
Total duration of critical care (days)		
Critical care survivors,^a^ median (IQR)	109	14 (6.1, 23)
Critical care non-survivors, median (IQR)	90	10 (5, 16)
Receipt and duration of organ support^2^		
Advanced respiratory support		
Receipt, *n* (%)	200	158 (79.0%)
Duration, median (IQR)	158	13 (8, 20)
Basic respiratory support *only*		
Receipt, *n* (%)	200	39 (19.5%)
Duration, median (IQR)	39	3 (2, 5)
Advanced cardiovascular support		
Receipt, *n* (%)	200	81 (40.5%)
Duration, median (IQR)	81	4 (2, 6)
Basic cardiovascular support *only*		
Receipt, *n* (%)	200	113 (56.5%)
Duration, median (IQR)	113	10 (5, 18)
Renal support		
Receipt, *n* (%)	200	62 (31.0%)
Duration, median (IQR)	62	7 (4, 15)
Neurological support		
Receipt, *n* (%)	199	21 (10.6%)
Duration, median (IQR)	21	4 (1, 6)

a One patient still receiving critical care was excluded. Total duration of critical care for this patient at the end of follow-up was 70 days.

b Duration of organ support is recorded as number of calendar days (00:00–23:59) on which support was received at any time, in those who received that type of organ support.

Organ supports are defined according to Critical Care Minimum Data Set^6^ as: Advanced respiratory support: invasive ventilation, BPAP via trans-laryngeal tube or tracheostomy, CPAP via trans-laryngeal tube, extracorporeal respiratory support; Basic respiratory support: >50% oxygen by face mask, close observation due to potential for acute deterioration, physiotherapy/suction to clear secretions at least two-hourly, recently extubated after a period of mechanical ventilation, mask/hood CPAP/BPAP, non-invasive ventilation, CPAP via a tracheostomy, intubated to protect airway; Advanced cardiovascular support: multiple IV/rhythm controlling drugs (at least one vasoactive), continuous observation of cardiac output, intra-aortic balloon pump, temporary cardiac pacemaker; Basic cardiovascular support: central venous catheter, arterial line, single IV vasoactive/ rhythm controlling drug; Renal support: acute renal replacement therapy, renal replacement therapy for chronic renal failure where other organ support is received; Liver support: management of coagulopathy and/or portal hypertension for acute on chronic hepatocellular failure or primary acute hepatocellular failure; and Neurological support: central nervous system depression sufficient to prejudice airway, invasive neurological monitoring, continuous IV medication to control seizures, therapeutic hypothermia.

In Table 3, we present critical care outcome, total duration of critical care, and receipt and duration of organ support (advanced respiratory, advanced cardiovascular and renal) in critical care for the first 200 consecutive patients compared with the figures previously published in ICNARC's weekly reports (results from alternate weeks' reports are presented). Over the sixteen weeks of ICNARC reporting, critical care mortality started higher than, increased and then decreased to a rate 5% lower than the rate for the first 200 consecutive patients. The reported total duration of critical care, for both survivors and non-survivors increased over time, from a median of 3 days to 12 days for survivors and a median of 3 days to 9 days for non-survivors, both lower than those reported for the first 200 consecutive patients, 15 and 10 days, respectively. Receipt and duration of organ support increased to values similar to those for the first 200 consecutive patients.

**Table 3.** Comparison of critical care survival, total duration of critical care and receipt and duration of organ support in critically ill patients with confirmed COVID-19 with weekly reports on early data.

Report date	20 March 2020	4 April 2020	17 April 2020	1 May 2020	15 May 2020	29 May 2020	12 June 2020	26 June 2020	10 July 2020	First 200
N with outcome/total	33/196	690/2249	2936/5578	5139/7542	6860/8699	8062/9347	8891/9777	9505/10,130	9995/10,421	199/200
Critical care outcome										
Critical care mortality, %	48.5	49.9	51.6	48.6	45.8	43.2	41.6	40.9	40.1	45.2
Total duration of critical care (days)										
Survivors, median (IQR)	3 (1, 5)	4 (2, 8)	5 (2, 9)	6 (3, 13)	9 (4, 19)	11 (4, 22)	11.5 (4,25)	12 (5, 26)	12 (5, 27)	15 (6, 23)
Non-survivors, median (IQR)	3 (1.5, 6)	5 (3, 8)	6 (4, 10)	7 (4, 13)	8 (5, 14)	9 (5, 15)	9 (5, 15)	9 (5, 16)	9 (5, 16)	10 (5, 16)
Receipt and duration of organ support^a^										
Advanced respiratory support										
Receipt, %	33.3	67.2	65.4	69.8	71.8	72.2	72.6	72.5	72.2	79
Duration, median (IQR)	5 (2, 7)	6 (4, 9)	7 (4, 11)	9 (5, 15)	11 (6, 18)	12 (7, 20)	13 (7,21)	13 (7, 22)	13 (7, 23)	13 (8, 20)
Advanced cardiovascular support										
Receipt, %	18.2	24.8	25.4	27.8	28.2	28.5	29.1	29.6	29.8	40.5
Duration, median (IQR)	3 (1, 5)	3 (1, 5)	3 (1, 5)	3 (1, 5)	3 (2, 6)	3 (2, 6)	3 (2, 6)	3 (2, 6)	3 (2, 6)	4 (2, 6)
Renal support										
Receipt, n (%)	12.1	18.5	20.3	23.1	24.6	25.2	26	26.4	26.6	31
Duration, median (IQR)	5 (3, 7)	4 (2, 6)	4 (3, 7)	5 (3, 10)	6 (3, 12)	7 (3, 13)	7 (3, 14)	7 (3, 14)	7 (3, 14)	7 (4, 15)

a Duration of organ support is recorded as number of calendar days (00:00–23:59) on which support was received at any time, in those who received that type of organ support.

Organ supports are defined according to Critical Care Minimum Data Set^6^ as: Advanced respiratory support: invasive ventilation, BPAP via trans-laryngeal tube or tracheostomy, CPAP via trans-laryngeal tube, extracorporeal respiratory support; Advanced cardiovascular support: multiple IV/rhythm controlling drugs (at least one vasoactive), continuous observation of cardiac output, intra-aortic balloon pump, temporary cardiac pacemaker; Renal support: acute renal replacement therapy, renal replacement therapy for chronic renal failure where other organ support is received.

## Discussion

COVID-19 in critical care is a disease with high mortality. Readmission to critical care occurred for 4.0% of patients and 5.5% died in hospital after discharge from critical care (10.1% of critical care survivors). COVID-19 places a large burden on critical care resources in terms of total duration of stay and provision of organ support, particularly advanced respiratory support. Early, weekly reported data by ICNARC did not fully reflect this burden.

ICNARC was well placed to rapidly collate, analyse and report data on patients critically ill with confirmed COVID-19 by virtue of its co-ordination of the CMP, the national clinical audit for adult critical care covering England, Wales and Northern Ireland. ICNARC built on lessons learned from the H1N1 pandemic, where response was too slow.^11,12^ While data collection, submission, analysis and reporting processes were speeded up to support timely information, data items were restricted to those routinely collected as part of the CMP. Feedback from clinical staff in critical care units indicated that the weekly information provided by ICNARC, in its reports, was used, locally, as the basis for discussions with patients and families and to understand the clinical care and outcomes in close to real time. More generally, the information underpinned the discussions across formal and informal networks of clinicians to facilitate understanding and learning about this new disease.

The UK is almost unique in producing critical care data so rapidly during this epidemic (as of 10 July 2020, on 12,793 admissions from 289 critical care units).^13^ In this study, patients were followed up until death or discharge from hospital, or, if still in hospital, for a minimum of 60 days from date of admission to critical care, to yield a representative and unselected cohort of patients critically ill with confirmed COVID-19. Completeness of outcomes compared favourably with other national^14^ and international reports.^15–17^ Receipt of organ support were broadly similar to previous international reports,^15,18,19^ except for the proportion of patients receiving renal support (31.0%), which was higher.^19–23^ We defined and collected only serious comorbidities, rather than any comorbidities, and therefore, report a lower proportion of patients with comorbidities (9.0%) compared with other reports.^15–17^

Early data, as an epidemic emerges, are important. With respect to critically ill patients with confirmed COVID-19, lower critical care mortality among patients with longer duration of critical care, indicated by the relatively flat shape of the Kaplan–Meier survival curve beyond 28 days, produced a bias towards higher estimates of mortality and shorter duration of organ support, early in the course of the epidemic. In an epidemic, where the demand for early data to inform the planning of services, both centrally and locally, needs to be balanced against the time and resources required for statistical modelling, approaches to mitigate any biases in the data remains a challenge.

## Conclusions

COVID-19 in critical care is a disease with high mortality that places a large burden on critical care resources. Early, weekly reported data by ICNARC did not fully reflect this burden. While early data as an epidemic emerges are important for clinicians and policymakers, careful consideration is needed in their interpretation, particularly for future planning.
